# *Arabidopsis thaliana* PRR7 Provides Circadian Input to the CCA1 Promoter in Shoots but not Roots

**DOI:** 10.3389/fpls.2021.750367

**Published:** 2021-10-15

**Authors:** Hugh G. Nimmo, Janet Laird

**Affiliations:** Institute of Molecular, Cell and Systems Biology, University of Glasgow, Glasgow, United Kingdom

**Keywords:** Arabidopsis, circadian, light dark cycles, luciferase imaging, PRR7

## Abstract

The core of the plant circadian clock involves multiple interlocking gene expression loops and post-translational controls along with inputs from light and metabolism. The complexity of the interactions is such that few specific functions can be ascribed to single components. In previous work, we reported differences in the operation of the clocks in Arabidopsis shoots and roots, including the effects of mutations of key clock components. Here, we have used luciferase imaging to study *prr7* mutants expressing CCA1::LUC and GI::LUC markers. In mature shoots expressing CCA1::LUC, loss of PRR7 radically altered behaviour in light:dark cycles and caused loss of rhythmicity in constant light but had little effect on roots. In contrast, in mature plants expressing GI::LUC, loss of PRR7 had little effect in light:dark cycles but in constant light increased the circadian period in shoots and reduced it in roots. We conclude that most or all of the circadian input to the CCA1 promoter in shoots is mediated by PRR7 and that loss of PRR7 has organ-specific effects. The results emphasise the differences in operation of the shoot and root clocks, and the importance of studying clock mutants in both light:dark cycles and constant light.

## Introduction

Circadian clocks have evolved in many organisms in response to the daily rotation of the earth and the resulting light:dark (LD) cycle. They drive rhythms at the molecular and cellular levels, regulate the timing of many aspects of physiology and behaviour and thus provide a fitness benefit (Green et al., 2002; Dodd et al., 2005). About one-third of the Arabidopsis genome is under circadian regulation (Michael and McClung, 2003; Covington et al., 2008). The circadian clock can influence plant processes at multiple levels from cell division to interaction with the environment (Fung-Uceda et al., 2018; Hubbard et al., 2018): at the physiological level, it can control such processes as photosynthesis, leaf movement, hormone responses, stem extension and stomatal opening (McClung, 2006; Harmer, 2009; Pruneda-Paz and Kay, 2010; Greenham and McClung, 2015).

In *Arabidopsis thaliana*, the core circadian oscillator includes multiple interlocking feedback loops of gene expression, modulated by post-translational control at several levels (Harmer, 2009; Nohales and Kay, 2016; McClung, 2019; Sanchez et al., 2020; Yan et al., 2021). The first loop to be discovered comprised the morning-expressed MYB transcription factors CIRCADIAN CLOCK ASSOCIATED 1 (CCA1) and LATE ELONGATED HYPOCOTYL (LHY) and the evening-phased transcriptional repressor PSEUDO-RESPONSE REGULATOR 1 (PRR1, also known as TIMING OF CAB EXPRESSION 1, TOC1). Other key players include the day-phased transcriptional repressors PRR9, PRR7 and PRR5, the evening-phased components EARLY FLOWERING 3 (ELF3), ELF4 and LUX ARRHYTHMO (LUX) which interact to form a transcriptional repressor named the evening complex (Huang and Nusinow, 2016), and CCA1 HIKING EXPEDITION (CHE). Several components, including REVEILLE 8 (RVE8), the NIGHT LIGHT-INDUCIBLE AND CLOCK-REGULATED (LNK) proteins and the LIGHT-REGULATED WD (LWD) proteins, provide positive arms of the clock.

The numerous components of the clock are arranged in a complex set of gene expression loops with multiple interactions, with additional inputs from factors, such as light and metabolism. The expression of CCA1 provides an example. It is negatively regulated by CCA1 itself, by LHY, by PRR5, 7 and 9, and by a combination of TOC1 and CHE (Sanchez et al., 2020). It is also activated by light; this involves the phytochrome signalling proteins FAR-RED ELONGATED HYPOCOTYL 3 (FHY3) and FAR-RED IMPAIRED RESPONSE 1 (FAR1) as activators, and TOC1 and PHYTOCHROME INTERACTING FACTOR5 (PIF5) as inhibitors (Liu et al., 2020). The PRRs and TOC1 have many targets in common. For example, genes, such as CCA1, LHY, LNKs, PIFs and RVE8, can be bound by two or more of PRR5, 7 and 9, and TOC1 (Nakamichi, 2020). PRR7 binds to numerous gene promoters *via* a G-box-like motif (Liu et al., 2013, 2016). The complexity of the interactions is such that few specific functions can be ascribed to single components, though PRR7 alone is responsible for the metabolic feedback to the clock by sugar levels (Haydon et al., 2013).

Recent work has provided ample evidence that different parts of a plant can oscillate with different free-running periods (Endo, 2016). This could result from differences in the wiring of the clock network and/or in the sensitivity of the clock to environmental inputs. For example, roots have a longer free-running period than shoots owing to differences in light inputs and the presence of metabolic sugars (James et al., 2008; Bordage et al., 2016; Nimmo, 2018; Greenwood et al., 2019). At higher resolution, cells in the root tip have a shorter period than cells in the rest of the tissue (Gould et al., 2018). The root and shoot clocks differ in their responses to mutations in the evening complex (Nimmo et al., 2020). Several mechanisms may contribute to coordination of the clock in the whole plant, including sugar signalling, long distance signals and light piping (Haydon et al., 2013; Takahashi et al., 2015; Nimmo, 2018). Given the importance of PRR7 in sugar signalling, we have studied the effects of its loss on mature shoots and roots in both light:dark cycles (LD) and constant light (LL) using two different reporters. The data show that loss of PRR7 has opposite effects on the periods of the shoot and root clocks, and that PRR7 provides the main circadian input to the CCA1 promoter in shoots.

## Materials and Methods

### Plant Material and Growth

Seeds of Col-0 and *prr7-11* expressing CCA1::LUC were from the Nottingham Arabidopsis Stock Centre (stock numbers N2107707 and N2107709, respectively). The Col-0 and *prr7-3* lines expressing GI::LUC have already been described (Greenwood et al., 2019). All seeds were surface sterilised, stratified for 4days at 4°C and sown on 1.2% agar in 0.5 strength Murashige and Skoog (MS) medium adjusted to pH 5.7 in 120mm square vertical plates which were exposed to LD cycles (12h white light provided by fluorescent tubes, 110–130μmol.m^−2^.s^−1^, 12h dark) at 20°C. 10days after sowing, seedlings (two clusters of three plants per plate) were transferred to fresh plates in which the top 3cm of agar had been replaced with 1.8% agar and 2% charcoal in 0.5 strength MS medium, readjusted to pH 5.7 after addition of charcoal. After a further 11days, plants were sprayed with luciferin and the plates were sealed with new lids containing a black barrier which separates the shoot and root compartments and prevents cross-contamination of their signals (Bordage et al., 2016).

### Luciferase Imaging

Plants (3weeks old) were sprayed with 60mm D-Luciferin in 0.01% triton (300μl per plate). Plates were kept at 20°C and illuminated by equal intensities of blue and red light provided by LEDs (Luxeon Star 447nm and 627nm, respectively, total intensity 25μmol.m^−2^.s^−1^ unless stated otherwise); the root compartments were not covered (Bordage et al., 2016). Plants were imaged for two full days in LD cycles as specified in the text followed by 96h in either LL or constant dark (DD). Bioluminescence was detected using a Photek 225/18 Intensified CCD camera with a 16mm lens. The camera and LEDs were controlled using Photek IFS32 software. Images (15min) were recorded every 1.5h in photon counting mode, without any filters. Root and shoot regions were defined and luminescence data extracted using Photek IFS32 software. The luminescence for each time point was normalised to the average luminescence over the corresponding time-course.

### Data Analysis

Normalised time-courses from imaging were analysed using Biological Rhythm Analysis Software System at BioDare2 (biodare2.ed.ac.uk, Zielinski et al., 2014) using the data from 24 to 96h in constant conditions. Period and relative amplitude error (RAE) were analysed using the FFT-NLLS suite of programmes. Differences in period were assessed by student’s t-test.

## Results

### *prr7-11* Shoots Show Minimal Circadian Input to the CCA1 Promoter

**Figure 1:**
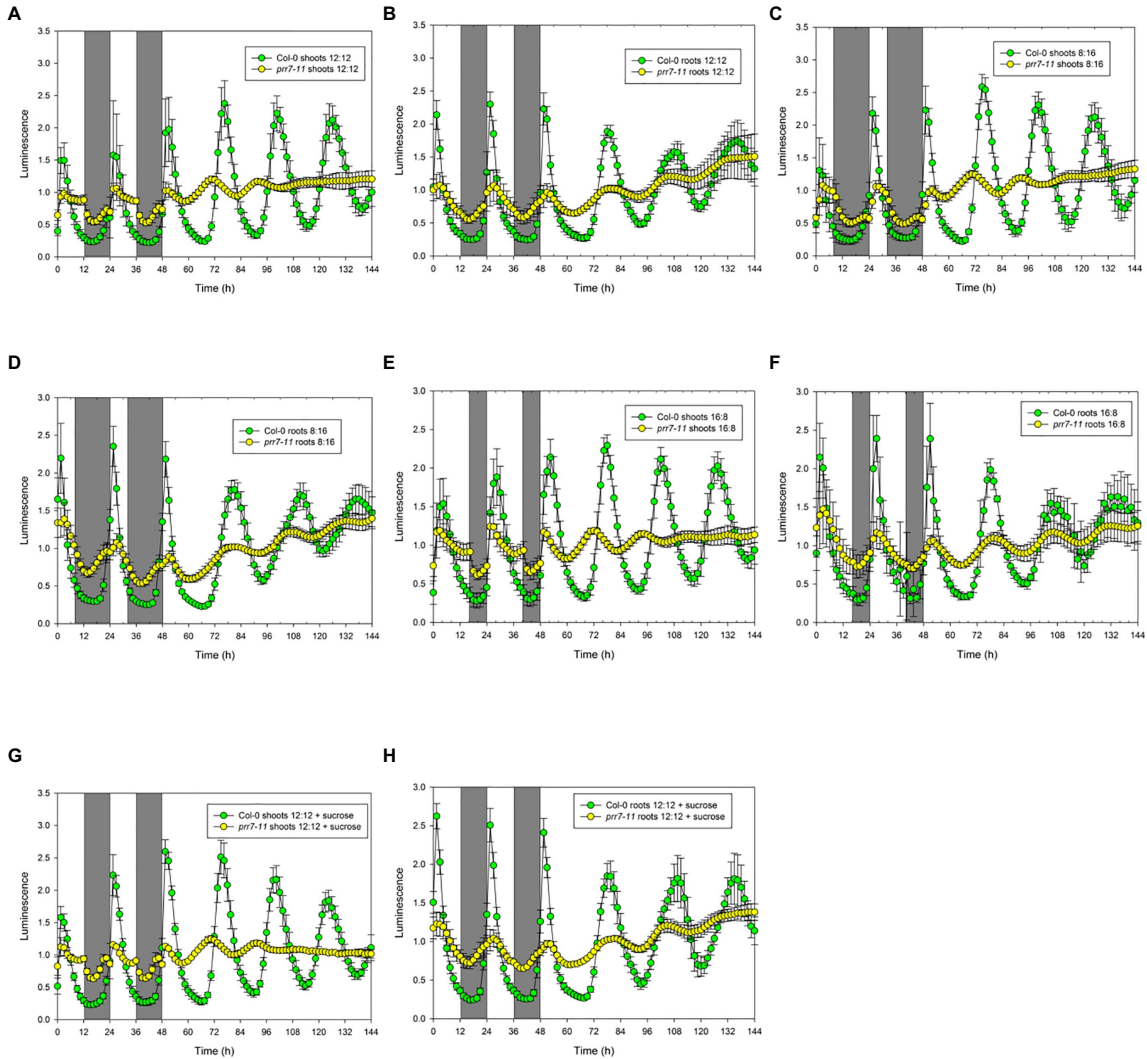
*prr7-11* shoots respond to light in LD cycles and are rhythmic in LL. The data show mean±SD luminescence for Col-0 and *prr7-11* shoots **(A,C,E,G)** and roots **(B,D,F,H)** expressing CCA1::LUC (n=16 replicates) in two LD cycles followed by 96h in LL. Photoperiods: **(A,B,G,H)** – 12:12; **(C,D)** – 8:16; and **(E,F)** – 16:8. Plates in **(G)** and **(H)** contained 1% sucrose. Green – Col-0; yellow – *prr7-11*.

The Arabidopsis lines *prr7-3* and *prr7-11* are non-functional mutants which carry the same T-DNA insertion in the first exon of the PRR7 gene; they were isolated from SALK_030430 by different groups (Michael et al., 2003; Yamamoto et al., 2003). We first assessed *prr7-11* expressing CCA1::LUC. We monitored luciferase activity over 2 d in LD cycles, using three different photoperiods, followed by 4 d in LL. Figure 1 shows that, at 25μmol/m^2^/s, the circadian clock in shoots and roots responds differently to the *prr7-11* mutation.

Col-0 shoots and roots both showed smooth curves of luciferase activity in 12h light: 12h dark followed by persistent rhythms in LL (Figures 1A,B). While *prr7-11* roots showed similar behaviour, albeit with low amplitude rhythms, *prr7-11* shoots in LD showed very abrupt transitions between light and dark, followed by transiently rhythmic behaviour in LL that died out after about 48h in LL. Essentially the same behaviour was seen where the LD cycle was either short day (8:16h) or long day (16:8h) (Figures 1C–F). Furthermore, the pattern was not affected by the presence of 1% sucrose (Figures 1G,H). This indicates that the abrupt transitions in LUC activity in *prr7-11* shoots at light:dark boundaries are not due to fluctuations in sucrose content.

**Figure 2:**
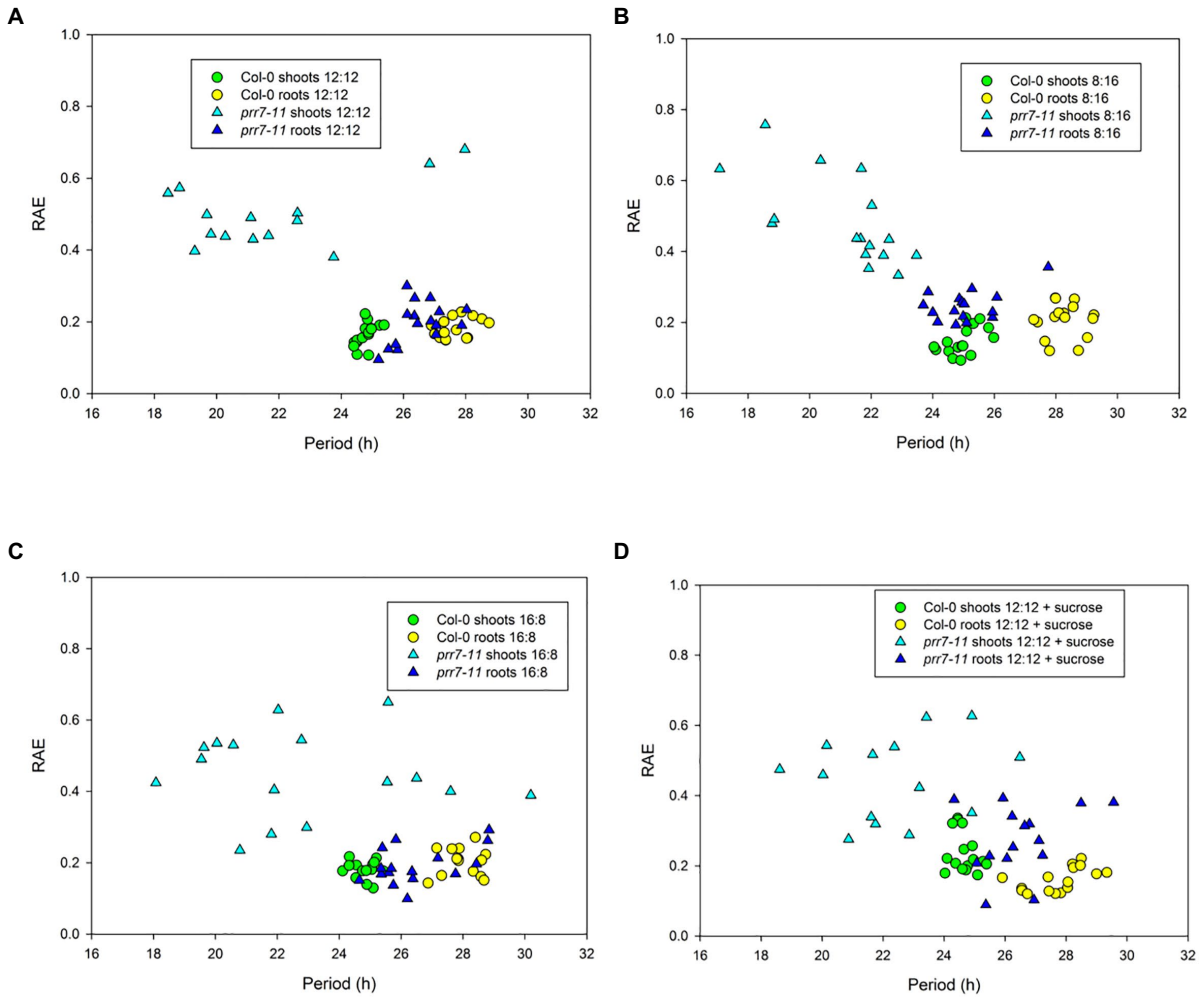
Period and robustness of rhythms in Col-0 and *prr7-11*. Period and RAE data for Col-0 (circles) and *prr7-11* (triangles) are from individual traces in the experiment shown in Figure 1. Photoperiods: **(A,D)** – 12:12; **(B)** – 8:16; and **(C)** – 16:8. Plates in **(D)** contained 1% sucrose.

To illustrate the robustness and period of rhythmic behaviour, Figure 2 presents plots of RAE against period for the same four conditions. In each case, Col-0 shoots and roots, and *prr7-11* roots, gave tightly clustered points indicative of robust and persistent rhythms. While over 75% of *prr7-11* shoot traces were scored rhythmic, the RAE values were high and the periods were very variable, indicating the lack of robust rhythmicity. The data in Table 1 show that *prr7-11* shortens the circadian period of roots in LL independent of the LD photoperiod, and in the presence of sucrose.

**Table 1:** *prr7-11* roots have a shorter period than Col-0 roots.

Photoperiod	Period ± SD (h)	Period ± SD (h)	*p* value
	Col-0 roots	*prr7-11* roots	
8:16	28.30±0.58	25.07±1.01	<0.0001
12:12	27.65±0.54	26.35±0.76	<0.0001
16:8	27.87±0.64	26.47±1.33	<0.001
12:12+sucrose	27.75±0.94	26.50±1.32	<0.01

The *p* values are for Col-0 *vs* prr7-11 roots by student’s *t*-test with *n*=16 replicates in all cases.

**Figure 3:**
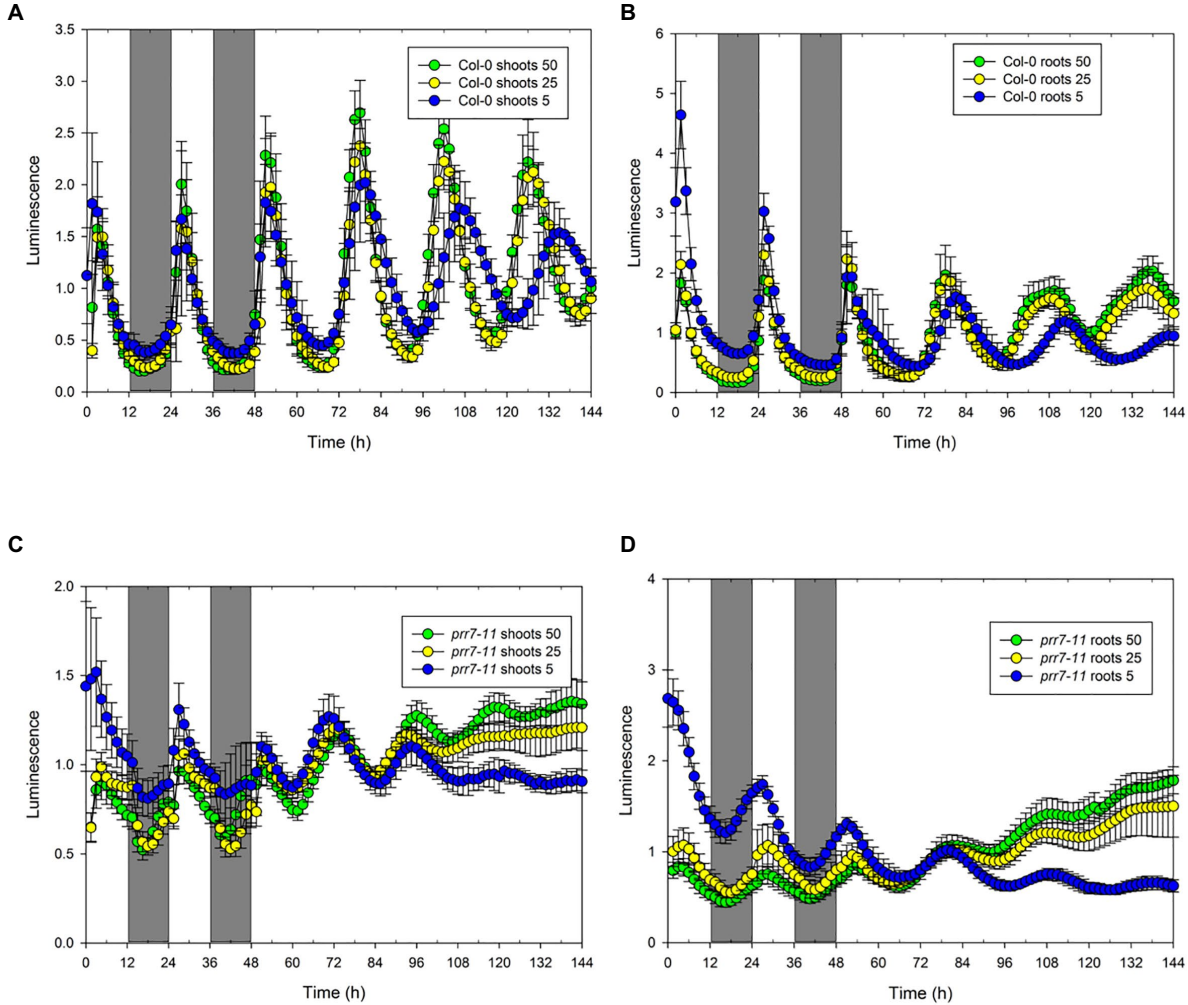
Effect of light intensity on rhythmic behaviour of Col-0 and *prr7-11*. The data show mean±SD luminescence for Col-0 (*n*=14; **A,B**) and *prr7-11* (*n*=18: **C,D**) shoots **(A,C)** and roots **(B,D)** expressing CCA1::LUC in two LD cycles followed by 96h in LL at the indicated light intensities in μmol/m^2^/s.

While the lack of effect of sucrose indicated that the abrupt transitions of LUC activity in *prr7-11* shoots were not due to photosynthetic production of sucrose, we tested the effects of different light intensities. Figure. 3 and Supplementary Table S1 show that the profiles of LUC activity in Col-0 shoots and roots, and in *prr7-11* roots, were very similar at 50 and 25μmol/m^2^/s, while at 5μmol/m^2^/s, the periods of the oscillations in LL were lengthened. This is compatible with the variation of period with light intensity shown in Nimmo (2018). In *prr7-11* shoots, illumination at 50μmol/m^2^/s somewhat increased the robustness of rhythms in LL but did not rescue the rhythmicity seen in Col-0 shoots. The abrupt transitions between light and dark were evident at 50μmol/m^2^/s but less so at 5μmol/m^2^/s. This suggested that there might be some residual circadian input to the CCA1 promoter in *prr7-11* shoots that is evident at very low light. We therefore tested behaviour over 2 d of LD cycles followed by constant dark (DD) in the presence of 1% sucrose to prolong rhythmicity. Supplementary Figure S1 shows that, as reported previously, DD rhythms persist longer in Col-0 roots than shoots. While low amplitude rhythmic behaviour persisted in *prr7-11* roots, this was not the case with shoots, confirming that there is little or no circadian input to the CCA1 promoter in *prr7-11* shoots.

### *prr7-3* Is a Long Period Mutant in Shoots but a Short Period Mutant in Roots

**Figure 4:**
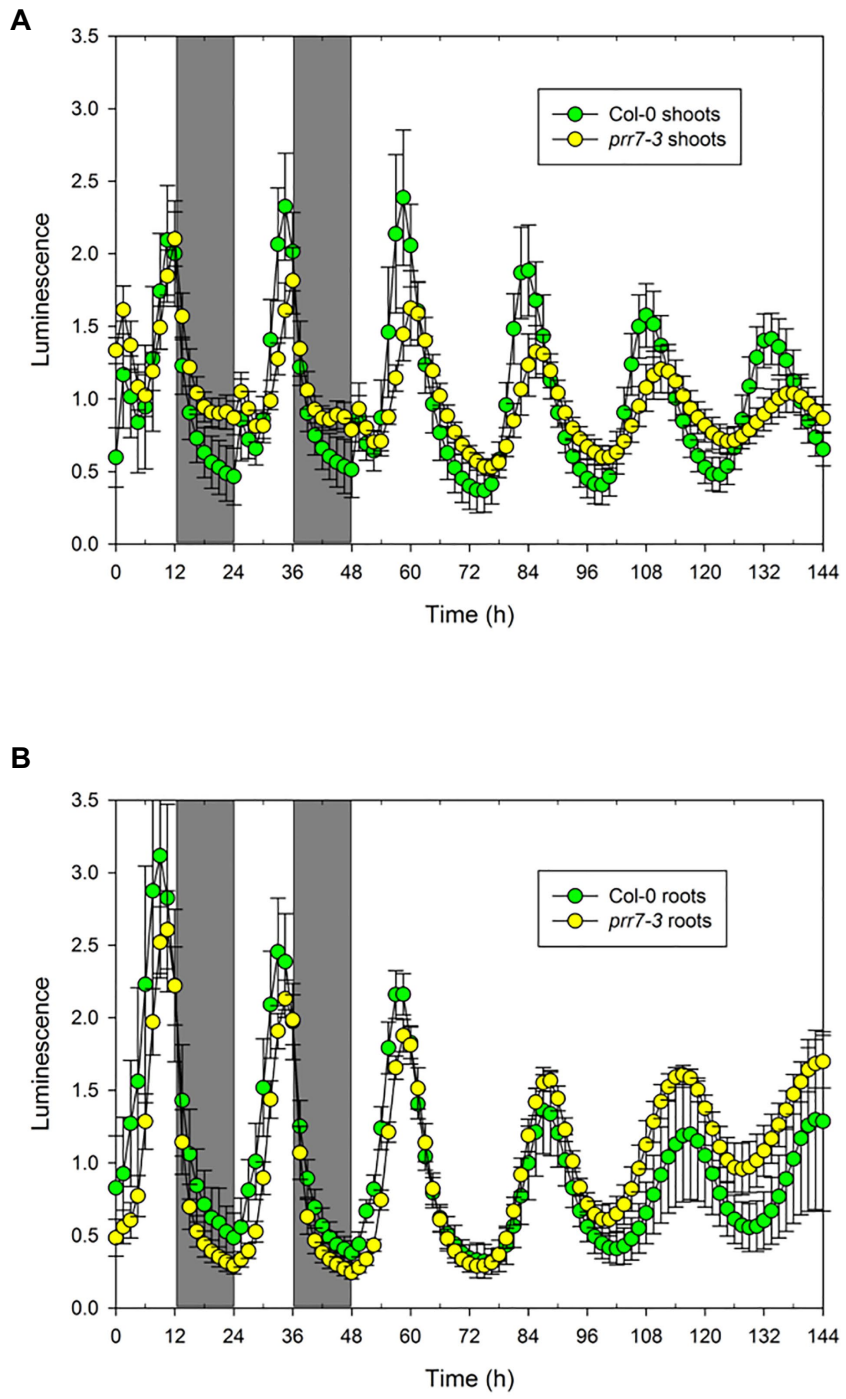
Rhythmicity in *prr7-3* shoots and roots. The data show mean±SD luminescence for Col-0 (*n*=28) and *prr7-11* (*n*=20) shoots **(A)** and roots **(B)** expressing GI::LUC in two LD cycles followed by 96h in LL at 25μmol/m^2^/s.

The data above show clearly that PRR7 is essential for circadian input to the CCA1 promoter in shoots, resulting in the arrythmicity of *prr7-11* shoots at lower light intensities. However, *prr7-11* roots retain rhythmicity, showing that this mutant retains a functional circadian clock with an altered period. We therefore tested *prr7-3* expressing GI::LUC. Figure 4 shows that in LD the shoots of Col-0 and *prr7-3* expressing GI::LUC behave similarly, with a small peak of LUC activity shortly after dawn but otherwise smooth rises and falls. In LL, *prr7-3* shoots and roots both maintain rhythmicity; in shoots, the period of *prr7-3* is longer than that of Col-0, whereas roots show the opposite behaviour (Supplementary Figure S2; Table 2). Thus for both markers, CCA1::LUC and GI::LUC, *prr7* mutation reduces the period of root rhythms. We also assessed the effect of *prr7* on phase. Supplementary Figure S3 shows that, with both markers, the circadian phase of Col-0 shoots (relative to subjective dawn) is earlier than that of roots, but this difference is much reduced or lost in *prr7* mutants.

**Table 2:** Differences in shoot and root periods between Col-0 and *prr7-3*.

Organ	Period ± SD (h)	Period ± SD (h)	*p* value
	Col-0	*prr7-11*	
shoots	24.59±0.57	25.27±0.80	<0.01
roots	28.22±0.55	27.39±0.62	<0.0001

The *p* values are for Col-0 *vs* prr7-3 by student’s *t*-test with *n*=28 replicates for Col-0 and *n*=20 replicates for prr7-3.

## Discussion

Our data show clearly that the effects of loss of PRR7 depend on the marker used. The lines *prr7-3* and *prr7-11* contain the same T-DNA insertion in the first exon (Farré et al., 2005). However, shoots of *prr7-11* expressing CCA1::LUC show almost square wave behaviour in LD cycles, with abrupt transitions between light and dark. This behaviour is seen at three different photoperiods and is barely affected by light intensities over the range 5–50μmol/m^2^/s. It is not affected by the presence of sucrose, so the abrupt transitions cannot be related to sugar signalling. In contrast, shoots of *prr7-3* expressing GI::LUC show a small peak of luciferase activity after dawn but do not show the abrupt transitions seen with *prr7-11* expressing CCA1::LUC. The peak of LUC activity shortly after dawn in the shoots of GI::LUC lines has been reported previously (Bordage et al., 2016) and is consistent with the small burst of GI mRNA after dawn observed by Locke et al. (2005). The dependence of phenotype in either LD cycles or LL on marker is unusual, though Palágyi et al. (2010) noted that the effects of *phyB-9* on period in LL depend on the marker used. The simplest explanation of these data is that in Col-0, the expression of CCA1 in LD cycles reflects both circadian and light inputs. PRR7 is essential for the circadian input in shoots, consistent with data showing that PRR7 represses CCA1 expression (Nakamichi et al., 2010). Given that several other circadian clock transcription factors bind to the same site in the CCA1 promoter as PRR7 (de los Reyes et al., 2020), it is possible that the circadian input involves multi-protein complexes of which PRR7 is an essential part. However, neither of the mutants *elf4* and *lux* expressing CCA1::LUC showed abrupt transitions in LD cycles (Nimmo et al., 2020), indicating that ELF4 and LUX are not essential for circadian input to CCA1 expression. Further work will be required to clarify the circadian input to the CCA1 promoter in shoots, and why PRR7 is not essential for this input in roots, for example studies of the effects of other mutations on light/dark transitions. In contrast to CCA1, PRR7 contributes to but is not essential for circadian input to the GI promoter in either shoots or roots.

In contrast to shoots, *prr7-11* has little effect on the pattern of CCA1::LUC expression in roots under LD cycles, though it does reduce amplitude. In particular, *prr7-11* roots do not show the abrupt transitions in luciferase activity in LD cycles that are observed in shoots. This suggests that light has much less effect on CCA1 expression in roots than in shoots. We have already shown that roots can detect light, including *via* light piping (Bordage et al., 2016; Nimmo, 2018), but it appears that this light detection in roots has little effect on the CCA1 promoter. Consistent with this, Bordage et al. (2016) showed that differences in light inputs are a major contributor to the difference between the shoot and root clocks. Both *prr7-11* and *prr7-3* roots retain robust rhythmicity, albeit with a shortened period. This shows that clock components other than PRR7 must contribute circadian input to the CCA1 and GI promoters in roots. A full understanding of differences between the shoot and root clocks will require assessment of the binding of clock components to clock genes in both organs.

The circadian phenotype of *prr7* mutants is usually reported as long period (Sanchez et al., 2020). However, Webb et al. (2019) suggested that this apparent phenotype may result from a difference in circadian plasticity between the wild type and *prr7*. In the wild type, period is dynamically adjusted in response to red light or sucrose, whereas in *prr7*, period is relatively rigid. Our data shows that, with GI::LUC as marker, *prr7-3* reduced circadian period in roots but increased it in shoots. Recently Li et al. (2020) reported similar results with a *prr7-3* line expressing CCA1::LUC through a split luciferase approach, and Greenwood et al. (2019) noted the reduced period of roots in *prr7-3*. We also found a reduction of root period in *prr7-11* expressing CCA1::LUC. In addition, using delayed fluorescence under low light conditions, Haydon et al. (2013) showed that *prr7-11* seedlings had a period some 2h shorter than that of Col-0. Overall, the effect of the *prr7* mutation on period clearly depends on the makeup of the circadian clock, which in turn depends on tissue, and on the experimental conditions used. This supports the view of Webb et al. (2019) that *prr7* mutations affect plasticity of the clock. The phase of the clock is also dynamically plastic (Webb et al., 2019). Our work shows that there is a phase difference between Col-0 shoots and roots for both CCA1::LUC and GI::LUC markers. This phase difference cannot be assessed in *prr7-11* because its shoots are not robustly rhythmic, but it is abolished in *prr7-3*. Thus, PRR7 seems to contribute to plasticity of phase as well as of period.

Most previous work on Arabidopsis clock mutants has used whole seedlings in LL. Our work depends on capture of luminescence from whole organs of mature plants. This approach conceals sub-tissue differences, such as the faster clock in root tips compared to the middle of the root (Gould et al., 2018; Greenwood et al., 2019). It will clearly be important to study the effects of *prr7* mutations at high spatial resolution. However, the data reported here provide important new information about the function of PRR7 in the plant clock and emphasise the importance of studying individual organs of mature plants in both LD and LL.

## Data Availability Statement

The datasets presented in this study can be found in online repositories. The names of the repository/repositories and accession number(s) can be found in the article/Supplementary Material.

## Author Contributions

HN conceived and designed the research, analysed the data, and wrote the paper. HN and JL performed the experiments. All authors contributed to the article and approved the submitted version.

## Funding

This work was supported by the Biotechnology and Biological Sciences Research Council grant BB/K006835/1 to HN.

## Conflict of Interest

The authors declare that the research was conducted in the absence of any commercial or financial relationships that could be construed as a potential conflict of interest.

## Publisher’s Note

All claims expressed in this article are solely those of the authors and do not necessarily represent those of their affiliated organizations, or those of the publisher, the editors and the reviewers. Any product that may be evaluated in this article, or claim that may be made by its manufacturer, is not guaranteed or endorsed by the publisher.
