# Supplementary material for: Arabidopsis thaliana PRR7 Provides Circadian Input to the CCA1 Promoter in Shoots but not Roots
Source: Front Plant Sci. 2021 Oct 15;12:750367. doi: 10.3389/fpls.2021.750367 (PMC8559795; doi:10.3389/fpls.2021.750367)
Supplement: Supplementary file 1 [file Data_Sheet_1.pdf]

Supplementary material

Arabidopsis thaliana PRR7 provides circadian input to the CCA1 promoter in shoots but not roots

Hugh G. Nimmo*, Janet Laird
Institute of Molecular, Cell and Systems Biology, University of Glasgow, Glasgow G12 8QQ, UK
*Corresponding author, email hugh.nimmo@glasgow.ac.uk

Figure S1 prr7-11 shoots are arrythmic in DD

A **B**

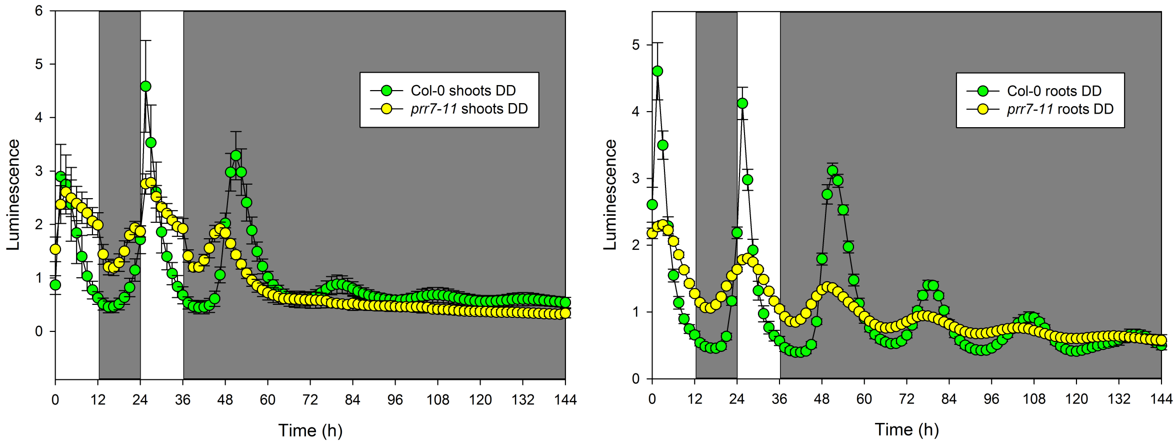

The data show mean ± SD luminescence for Col-0 and *prr7-11* shoots (**A**) and roots (**B**) expressing CCA1::LUC (n = 14 replicates) in two LD cycles followed by DD.

Figure S2 Period and robustness of rhythms in Col-0 and *prr7-3*

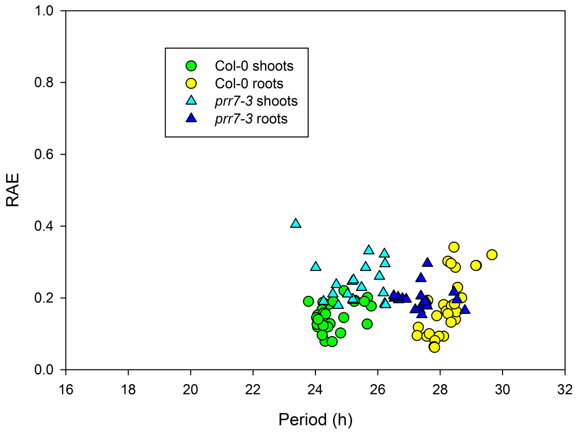

Period and RAE data for Col-0 (circles) and *prr7-3* (triangles) are from individual traces in the experiment shown in Fig. 4.

Figure S3 Effects of *prr7* mutation on phase

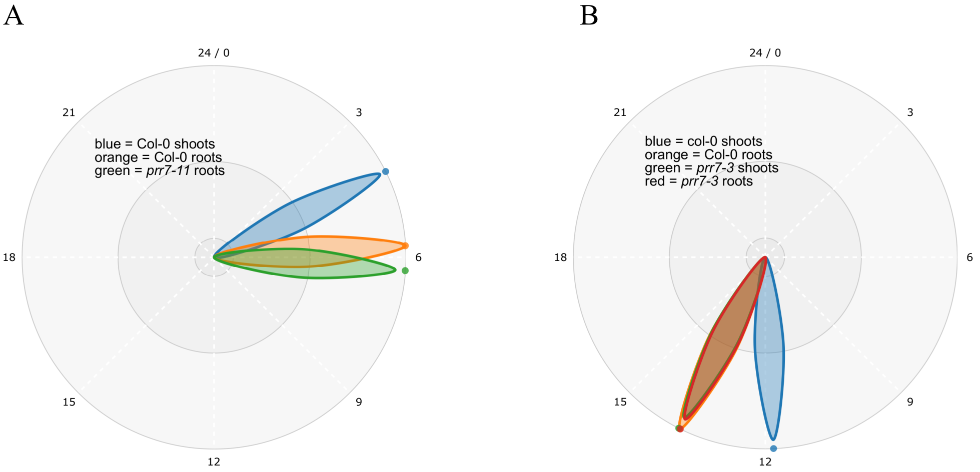

Average circadian phases were calculated relative to subjective dawn using the individual time courses in the experiments shown in Fig. 1A,B and Fig. 4A,B, see https://biodare2.ed.ac.uk/documents/phases.

Table S1 Effect of light intensity on period in Col-0 and *prr7-11*

Sample	Period ± SD (h)	Period ± SD (h)	P value
	Intensity 50 µmol/m^2/s	Intensity 5 µmol/m^2/s	
Col-0 shoots	24.40 ± 0.30	27.94 ± 0.41	<0.0001
Col-0 roots	28.21 ± 0.84	30.08 ± 0.54	<0.0001
prr7-11 roots	25.47 ± 1.45	29.24 ± 0.90	<0.0001

The P values are for 50 vs 5 µmol/m^2/s by Student's t test with n = 16 replicates in all cases. *prr7-11* shoots are not included because of their lack of robustness.

DATA AVAILABILITY

The data that support the findings of this study are available at biodare2.ed.ac.uk, experiment numbers 19235, 19437 and 19781.
